# Electrospun Scaffolds Based on Poly(butyl cyanoacrylate) for Tendon Tissue Engineering

**DOI:** 10.3390/ijms24043172

**Published:** 2023-02-06

**Authors:** Eleonora Bianchi, Barbara Vigani, Marco Ruggeri, Elena Del Favero, Caterina Ricci, Pietro Grisoli, Anita Ferraretto, Silvia Rossi, César Viseras, Giuseppina Sandri

**Affiliations:** 1 Department of Drug Sciences, University of Pavia, Viale Taramelli 12, 27100 Pavia, Italy; 2 Department of Medical Biotechnology and Translational Medicine, University of Milan, LITA Viale Fratelli Cervi 93, 20090 Segrate, Italy; 3 IRCCS Galeazzi Orthopaedical Institute, Laboratory of Experimental Biochemistry & Molecular Biology, Via R. Galeazzi 4, 20161 Milan, Italy; 4 Department of Biomedical Sciences for Health, University of Milan, LITA, Via Fratelli Cervi 93, 20090 Segrate, Italy; 5 Department of Pharmacy and Pharmaceutical Technology, Faculty of Pharmacy, University of Granada, Campus of Cartuja s/n, 18071 Granada, Spain

**Keywords:** tendon disorders, poly(butyl cyanoacrylate), copper oxide, caseinophosphopeptides, electrospinning, anti-inflammatory, antioxidant, antimicrobial

## Abstract

Tendon disorders are common medical conditions that could lead to significant disability, pain, healthcare costs, and a loss of productivity. Traditional approaches require long periods of treatment, and they largely fail due to the tissues weakening and the postoperative alterations of the normal joint mechanics. To overcome these limitations, innovative strategies for the treatment of these injuries need to be explored. The aim of the present work was the design of nano-fibrous scaffolds based on poly(butyl cyanoacrylate) (PBCA), a well-known biodegradable and biocompatible synthetic polymer, doped with copper oxide nanoparticles and caseinphosphopeptides (CPP), able to mimic the hierarchical structure of the tendon and to improve the tissue healing potential. These were developed as implants to be sutured to reconstruct the tendons and the ligaments during surgery. PBCA was synthetized, and then electrospun to produce aligned nanofibers. The obtained scaffolds were characterized for their structure and physico-chemical and mechanical properties, highlighting that CuO and CPP loading, and the aligned conformation determined an increase in the scaffold mechanical performance. Furthermore, the scaffolds loaded with CuO showed antioxidant and anti-inflammatory properties. Moreover, human tenocytes adhesion and proliferation to the scaffolds were assessed in vitro. Finally, the antibacterial activity of the scaffolds was evaluated using *Escherichia coli* and *Staphylococcus aureus* as representative of Gram-negative and Gram-positive bacteria, respectively, demonstrating that the CuO-doped scaffolds possessed a significant antimicrobial effect against *E. coli*. In conclusion, scaffolds based on PBCA and doped with CuO and CPP deserve particular attention as enhancers of the tendon tissue regeneration and able to avoid bacterial adhesion. Further investigation on the scaffold efficacy in vivo will assess their capability for enhancing the tendon ECM restoration in view of accelerating their translation to the clinic.

## 1. Introduction

Tendon disorders are common medical conditions that could lead to significant disability, pain, healthcare costs, and a loss of productivity. A wide range of injury mechanisms exist, such as tears, which can occur in healthy tendons that are acutely overloaded or lacerated, or tendinitis or tendinosis, which can occur in tendons exposed to overuse conditions or intrinsic tissue degeneration [1,2].

In general, the healing of tendons follows the typical wound healing course, including an early inflammatory phase, which lasts about one week, followed by a proliferative phase, which lasts from three to four weeks, and finally by a remodeling phase, which lasts many months. The exact duration of each phase is highly dependent on the subject, the tendon location, and the injury type. For this reason, it is of paramount importance to provide an adequate support to the tissue during the entire regeneration process [3,4].

Numerous treatment approaches have been attempted to improve tendon healing, including surgical approaches and cell-based therapies, with specific rehabilitation protocols. However, these require long periods of treatment, and they largely fail due to the tissues weakening and the postoperative alterations of the normal joint mechanics [5,6].

To overcome these limitations, in recent years several studies have been conducted for the application of tissue engineering in the treatment of orthopedic injuries, aiming at the regeneration of damaged tissues, instead of replacing them, through the development of reparative constructs. For this purpose, poly(butyl cyanoacrylate) (PBCA) represents an interesting candidate to develop innovative platforms, as it is a well-known biodegradable and biocompatible synthetic polymer, used nowadays in the medical field as a nanocarrier for drug delivery [7,8]. Considering these premises, the aim of the present work was the design of nano-fibrous scaffolds based on PBCA, and doped with copper oxide nanoparticles (CuO) and caseinophosphopeptides (CPP), able to mimic the hierarchical structure of the tendon and to improve the tissue healing potential. These were developed as implants to be sutured to reconstruct the tendons and the ligaments during surgery.

Inorganic nanomaterials have recently gained great attention in tissue engineering, in particular to dope polymeric scaffolds [9,10]. In fact, they have unique properties, such as antimicrobial, antioxidant, and anti-inflammatory properties, and thus they have been widely used to improve polymeric scaffold mechanical properties, and to support and enhance the cell growth [9,10]. In particular, in the tissue engineering field, CuO nanoparticles proved to stimulate cell proliferation during wound healing by upregulating the vascular endothelial growth factor gene expression, promoting mesenchymal stem cell differentiation, and avoiding infections via their antibacterial properties. Moreover, they were demonstrated to increase the physico-chemical properties of biomaterials, such as the mechanical strength [11,12]. CPPs—phosphorylated peptides enzymatically released from casein due to in vitro and in vivo digestion—have been loaded in the scaffolds since they were proven to promote calcium uptake, due to their cation-binding activity, osteoblast differentiation [13], and exertion of antioxidant activity [14], and for these reasons they seemed suitable candidates to potentiate the effectiveness of CuO-doped PBCA scaffolds.

The developed scaffolds should be able to mimic the hierarchical structure of the tendon after being sutured, consequently improving the tissue reconstruction and the healing potential. Moreover, they should protect the site of inflammation from severe tissue damage and enhance cell proliferation and migration. Finally, the scaffolds, the object of this study, should also protect the surgical site from bacterial infections, which could lead to complications, such as tissue destruction, failure or extension of proper wound healing, and occasionally bacteremia, resulting in prolonged hospital stays, and increased patient costs for the healthcare system [15].

## 2. Results and Discussion

### 2.1. Synthesis and Characterization of PBCA

The polymerization process of the BCA is characterized by a yield % of 80.02% ± 7.85%, and the molecular weight and the polymerization degree of the obtained polymer are 749 Da and 4.89, respectively.

**Figure 1:**
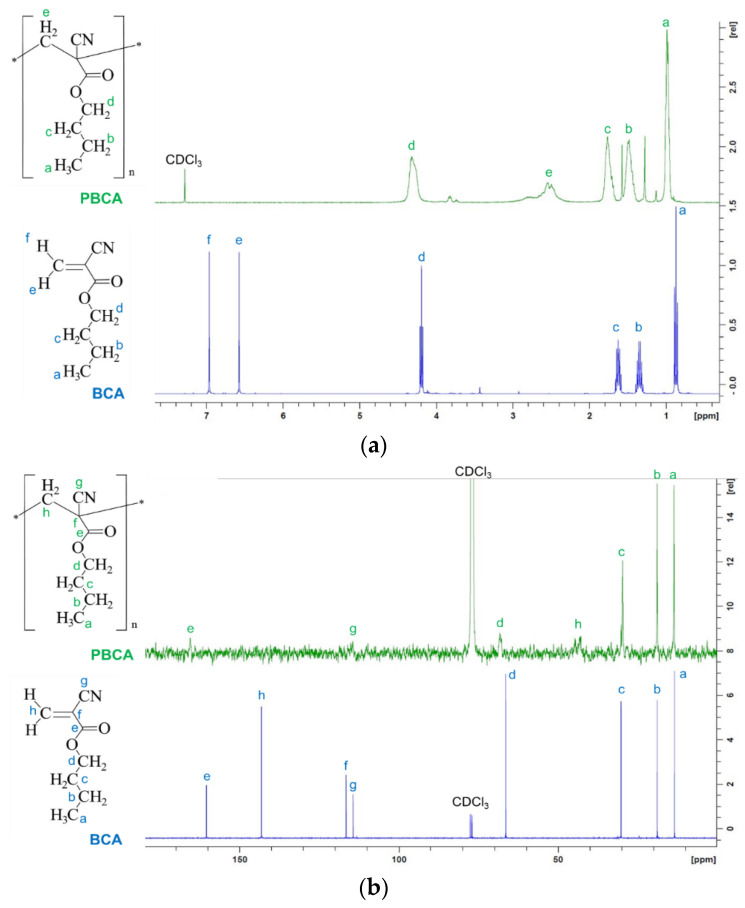
(**a**) ^1^H and (**b**) ^13^C NMR spectra of BCA and PBCA powders measured in deuterated chloroform (CDCl_3_).

Figure 1 reports the chemical shifts and the signal assignments of BCA and PBCAs ^1^H (Figure 1a) and ^13^C (Figure 1b) NMR spectra. Other spectra reported in the literature for this type of structure were taken as references [16] to make the assignments. Several specific signals with clearly distinct shift differences are observed in the ^1^H NMR spectra (Figure 1a). Proton signals corresponding to the broad multiplet of the α-methylene of the ester residue (-O–CH_2_–) are found at 4.12 ppm (d, blue and green signals), while the broad multiplets of β- and γ-methylene groups (–CH_2_–) are found at 1.62 and 1.41 ppm, respectively (b and c, blue and green signals). The broad triplet of the methyl group (–CH_3_) is found at 0.92 ppm (a, blue and green signals).

On the other hand, protons signals from the monomer vinyl group (at 7.0 and 6.63 ppm; e and f, blue signals) are not present in the spectra of PBCA, but a signal assigned to protons of the methylene group of the main chain of PBCA appears at 2.43 ppm (broad multiplets; e, green signal), confirming the monomer conversion into a polymer.

### 2.2. Scaffold Physico-Chemical Characterization

The PBCA optimal concentration to obtain fine nanofibers with the homogeneous morphology was 20% *w/w*. In particular, it is possible to observe that the PBCA concentration affects the fiber morphology. In fact, the increased concentration leads to the formation of irregular fibers, and knots. Moreover, the P3 blend, containing the higher polymer concentration, does not even allow to obtain a regular fiber production. On the other hand, the lower polymer concentration leads to the formation of uniform fibers with a smooth surface and nanometric dimensions (Figure S1). For this reason, the P1 blend was selected to be doped with CuO and CPP.

**Figure 2:**
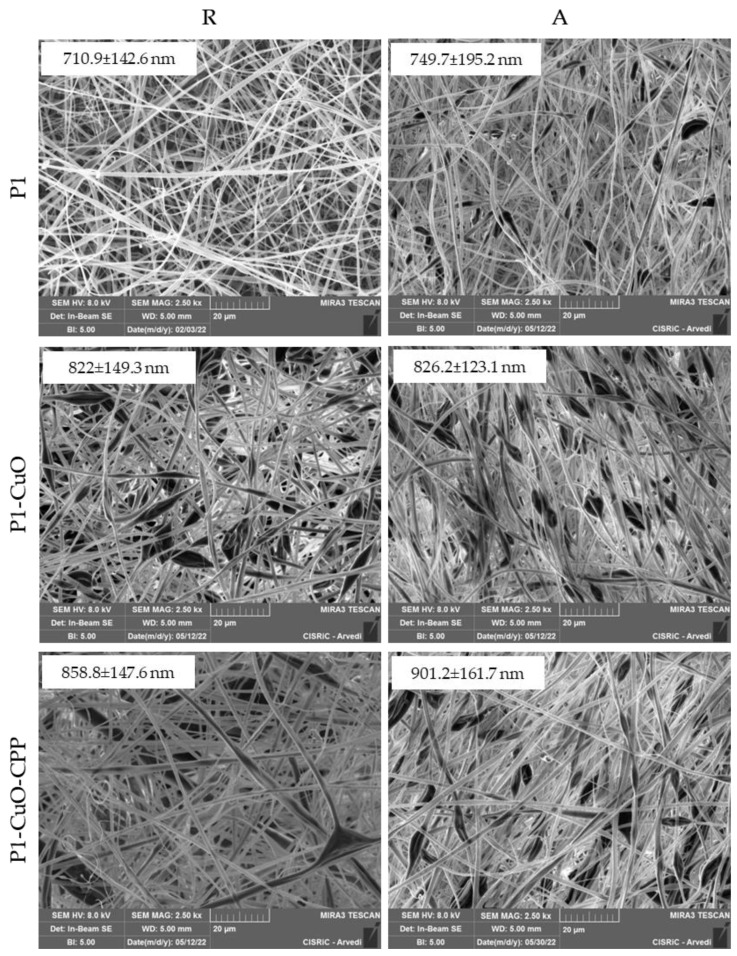
SEM micrographs of P1, P1-CuO, and P1-CuO-CPP scaffolds randomly collected (R) and aligned (A) at 2.5 kx magnification. In the insets, the corresponding dimensional analysis is shown (mean values ± SD, *n* = 30).

In Figure 2, the SEM micrographs of the random (R) and aligned (A) scaffolds are shown. The CuO and CPP doping leads to the formation of bulges, probably due to the aggregation of the nanoparticles (CuO) into the fiber structure. Despite this, the fibers maintain nano-dimensions. Moreover, the scaffolds obtained from the cylindrical collector, especially the ones loaded with the active components, shows an orderly spatial organization of the fibers.

**Figure 3:**
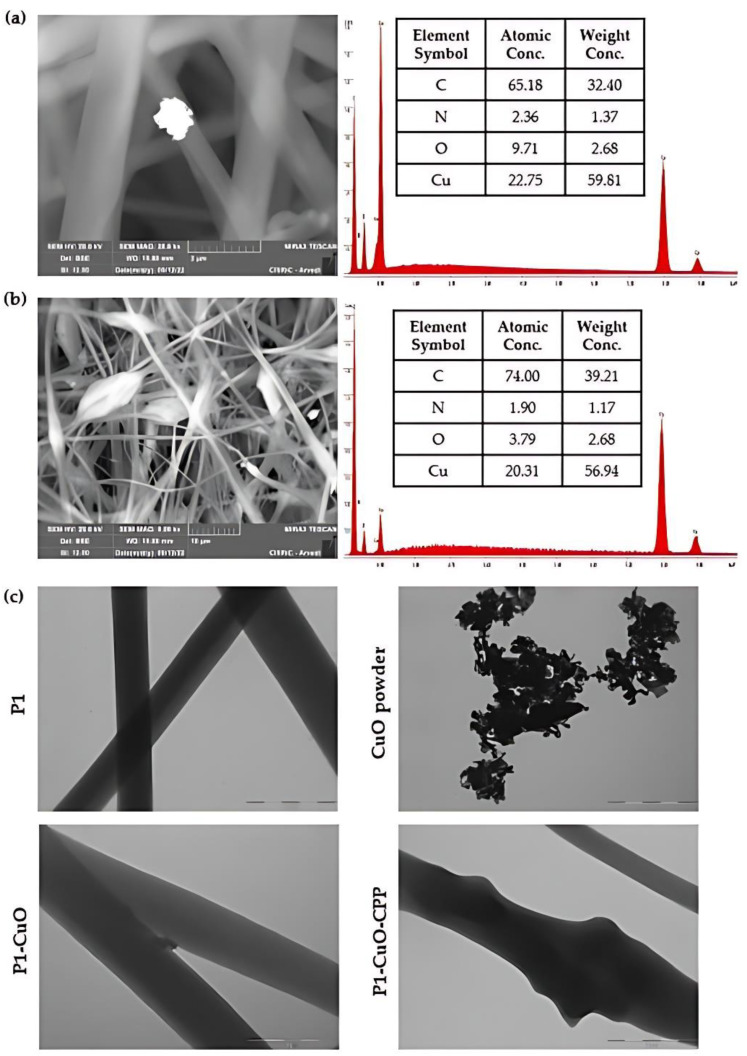
SEM-EDX of (**a**) P1-CuO and (**b**) P1-CuO-CPP scaffolds. (**c**) TEM micrographs of the scaffolds with and without CuO and of the CuO powder (scale bar: 1 µm).

SEM-EDX and TEM analyses (Figure 3) were performed to evaluate the CuO incorporation into the fibers. The EDX (Figure 3a,b) underlines the presence of CuO clusters both onto the fibers and into the fiber bulges. The same trend is noted in the TEM images (Figure 3c), in which it is possible to observe the empty fibers of polymer alone, and the same CuO clusters onto the fibers and into the fiber bulges, affecting their shape and the surface roughness. The presence of CPP is not visible in the fibers, suggesting that they are homogeneously dispersed and entangled with the PBCA chains.

The interfacial properties and wettability of the scaffolds, both in the aligned and random structures, were evaluated using contact angle measurements. The scaffold morphology and dimensions do not change after hydration.

**Figure 4:**
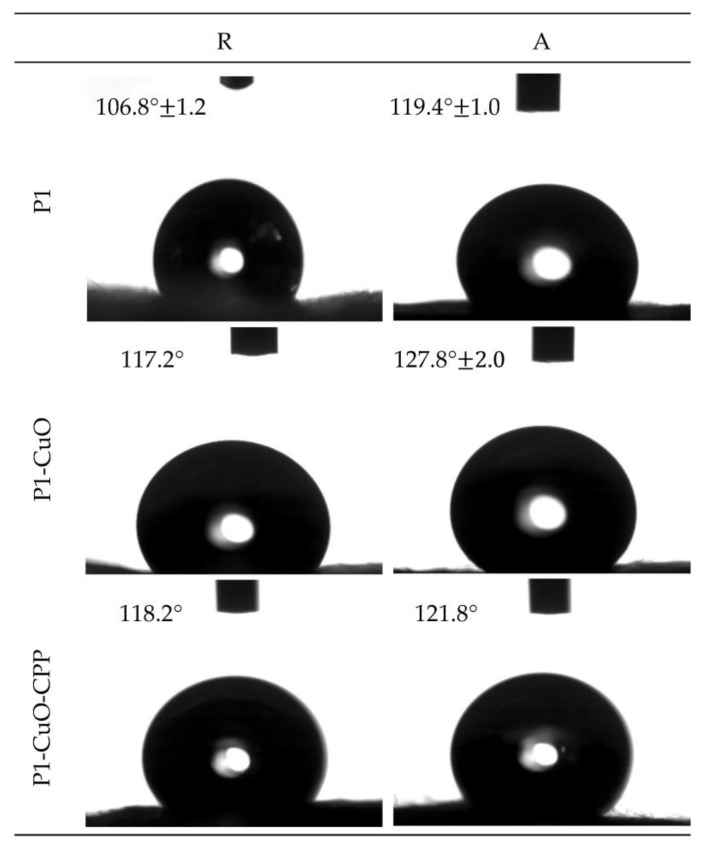
Images of the buffer drop onto the surface of P1, P1-CuO, and P1-CuO-CPP random (R) and aligned (A) scaffolds after 1000 ms. In each image, the value of the contact angle is reported (mean values ± SD, *n* = 4) (needle diameter: 0.405 mm). One-way ANOVA, Scheffé test (*p* ≤ 0.05) is reported in the Supplementary Information (SI: Section S1.3.1).

Figure 4 reports the shape and the contact angle values of a 0.4 µL buffer drop released onto the scaffolds.

It is possible to observe how the fiber orientation governs the wettability of the scaffolds, despite having the same polymeric matrix composition. In fact, the random scaffolds are characterized by contact angle values lower than the aligned ones. This could be attributable to the surface organization, which could influence the water spreading onto the scaffold surface. In particular, the inter-fiber spacing of the aligned nanofibers leads to a capillary-like force parallel to the fiber orientation, consequently preventing water spreading in the cross-direction. On the other hand, in random scaffolds, the forces induced by adjacent nanofibers are also randomly directed without any influence on hydration [17,18].

Furthermore, the presence of CuO nanoparticles into the fibrous matrix resulted in a significant increase in the contact angle of the scaffolds. This could be attributed to the hydrophobic nature of the CuO [19,20]. Moreover, the contact angle could also be affected by the surface roughness [20,21]. Therefore, these results are consistent with the increase in the surface roughness that characterizes the scaffolds containing CuO nanoparticles compared with the undoped ones.

### 2.3. Structural Characterization

**Figure 5:**
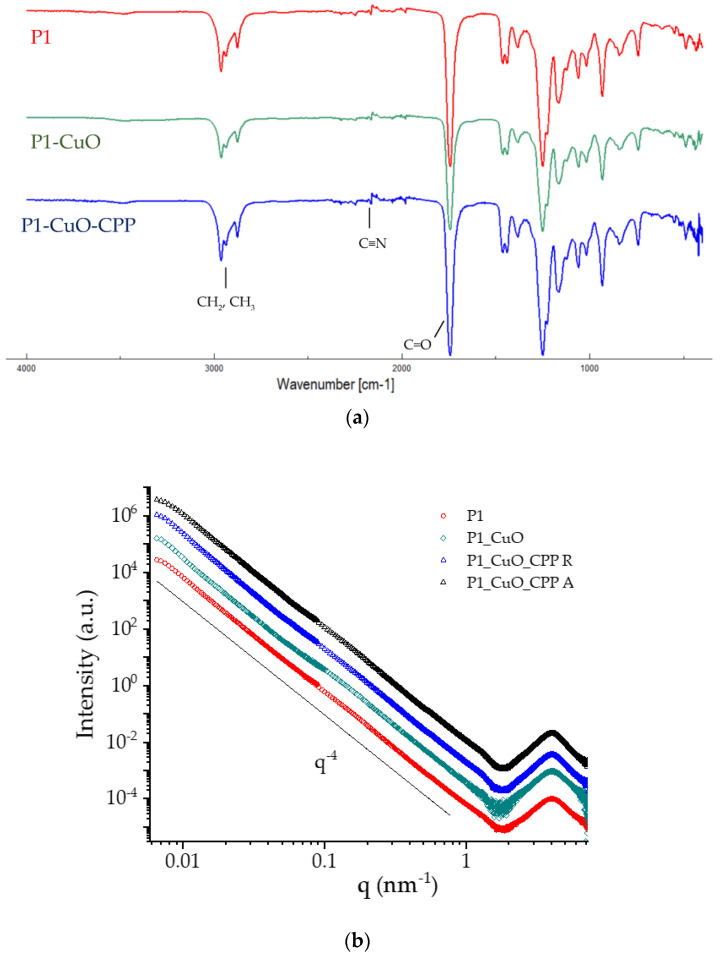
(**a**) FTIR spectra of P1, P1-CuO, and P1-CuO-CPP scaffolds. (**b**) SAXS spectra of P1, P1-CuO, and P1-CuO-CPP scaffolds randomly collected (R) and of P1-CuO-CPP aligned (A). The intensity profiles were vertically shifted for better visibility.

The structural characterization of the scaffolds was studied using infrared spectroscopy, and small-angle X-ray spectroscopy. Figure 5a reports the FTIR profiles of the electrospun scaffolds. All the spectra are characteristic of the PBCA, as it is the main component of the systems (Figure S2). In fact, the bands at 1747 and 1253 cm^−1^ suggest the presence of ester groups, while the one at 2237.49 cm^−1^ is due to the stretching of the cyano group. Moreover, the absence of bands related to the stretching vibration of the vinyl group’s C=C bond, usually found at 1675 cm^−1^, as well as the absence of an absorption band at 3010 cm^−1^, usually associated with the C–H of vinyl groups, ulteriorly confirm the polymerization reaction efficiency [22].

SAXS measurements were performed on scaffolds just after insertion in capillaries and full wetting to obtain structural information on the nanoscale. The SAXS spectra (I(q) vs. q) are reported in Figure 5b in the whole SAXS range, vertically shifted for better visibility. All spectra show a broad peak in the high-q region, at q = 4 nm^−1^, corresponding to a characteristic distance of d = 1.6 nm, due to the very local arrangement of the polymer chains within the fibers. In the region q < 1 nm^−1^, the intensity profiles follow a (q^−4^)-slope over a large q-range, covering the micro-to-nanoscale, typical of compact objects with smooth surfaces. The spectra of scaffolds in the presence of CuO nanoparticles or CPPs and CuO nanoparticles are similar to the naked one. This is conceivably due to the low-doping doses of CuO nanoparticles (1%) and CPPs (0.5%) in the fibrous matrix that does not affect the local packing of polymer chains and the compactness of the surface on the nanoscale, similarly for random and aligned scaffolds. The spectra of the two doping components are reported in Figure S3. Results indicate that CuO nanoparticles have a rod-like shape (short size = 10 nm, long size = 70 nm), while CPPs behave as gaussian polymer chains.

### 2.4. Mechanical Properties

**Figure 6:**
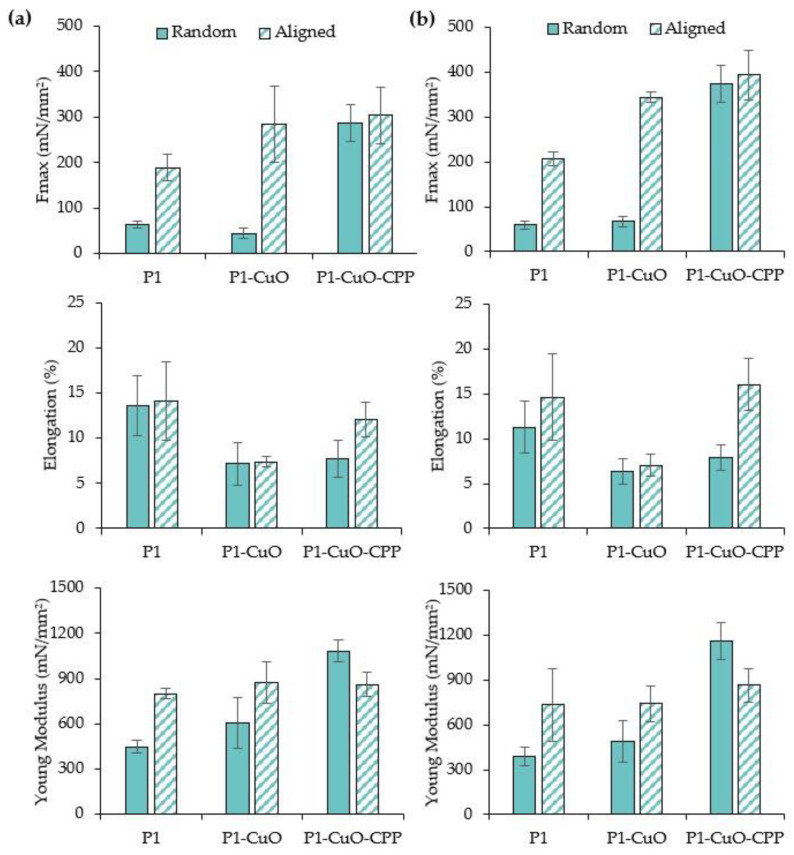
Maximum tensile force values (Fmax), elongation (%), and Young’s modulus (mN/mm^2^) evaluated for P1, P1-CuO, and P1-CuO-CPP random (full color) and aligned (shaded color) scaffolds in both (**a**) dry and (**b**) hydrated states (mean values ± SD, *n* = 5). One-way ANOVA, Scheffé test (*p* ≤ 0.05) is reported in the Supplementary Information (SI: Section S1.3.2).

The mechanical properties of both random and aligned scaffolds were evaluated in both dry (Figure 6a) and hydrated (Figure 6b) states.

The aligned structure and the doping with the active components lead to a significant increase of the maximum force at break (Fmax) values, while the elongation is lower than those of the undoped scaffolds. This is principally due to the presence of the CuO nanoparticles in the fibrous matrix. In fact, the mechanical properties could be influenced by the nanoparticle distribution into the fibrous structure. In particular, they may have reduced the flexibility of the scaffolds by decreasing the free volume between the polymer chains and, consequently, their mobility [19]. The fiber orientation also plays a pivotal role in the mechanical performance since the aligned scaffolds are characterized by Fmax values significantly higher than the random ones. In fact, previous studies also highlighted that the mechanical properties could be affected by the angle between the stretching direction and the fiber orientation, significantly increasing when this was 0° [23].

### 2.5. DPPH Radical Scavenging Activity

Since an enhanced generation of radical oxygen species (ROS), produced by neutrophils and macrophages, at the site of inflammation could cause severe tissue damage and impair cell proliferation and migration, a scaffold with antioxidant properties could represent an important tool to effectively tackle chronic lesions. In fact, the antioxidants could guarantee the balance between the favorable ROS properties, such as angiogenesis stimulation, and the negative ones (oxidative stress and healing delay).

**Figure 7:**
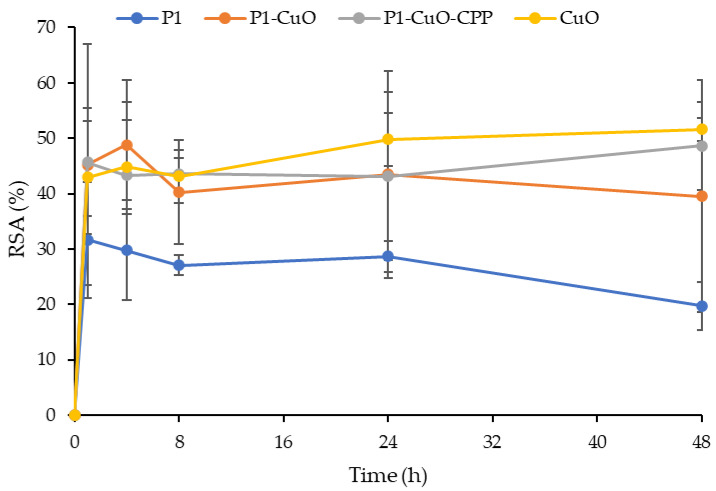
Radical scavenging activity (RSA%) evaluated via the DPPH assay for the P1, P1-CuO, and P1-CuO-CPP scaffolds and for the CuO and CPP powders alone (mean values ± SD, *n* = 5). One-way ANOVA, Scheffé test (*p* ≤ 0.05) is reported in the Supplementary Information (SI: Section S1.3.3).

For this reason, these properties were characterized via the DPPH assay, evaluating the capability of the scaffolds to reduce DPPH, a standard nitrogen-concentrated free radical. Figure 7 reports the antioxidant properties of the scaffolds and the CuO powder alone as radical scavenging activity (RSA%). The results show that the incubation time of the scaffolds doped with CuO nanoparticles in PBS decreased the DPPH%, suggesting that the antioxidant activity increases over time, probably due to the release of bioactive molecules. In fact, as reported by Das et al., CuO nanoparticles have a capability to transfer their electron density towards the free radical located at the nitrogen atom in DPPH [24].

### 2.6. Cell Adhesion and Proliferation

**Figure 8:**
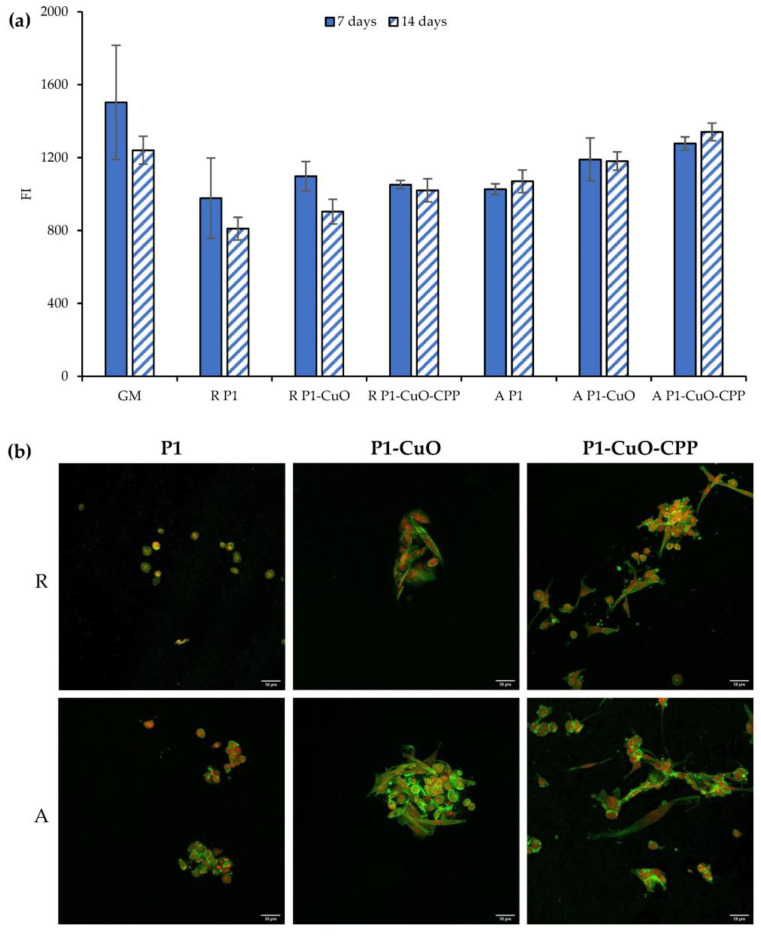
(**a**) Alamar Blue assay evaluated for P1, P1-CuO, and P1-CuO-CPP scaffolds, both random (R) and aligned (A), at 7 and 14 days of culture, compared to the cells grown in standard conditions (GM). (**b**) CLSM images of TEN-1 cells grown onto P1, P1-CuO, and P1-CuO-CPP scaffolds, random (R) and aligned (A), at 14 days of culture (nuclei stained in red, cytoskeleton stained in green) (mean values ± SD, *n* = 5). One-way ANOVA, Scheffé test (*p* ≤ 0.05) is reported in the Supplementary Information (SI: Section S1.3.4).

In view of the application of aligned scaffolds in the repair of the tendon interface, TEN-1 cells were selected as representatives for tendon cells, and they were grown onto the scaffolds, both random and aligned, to evaluate the influence of the fiber conformation onto the cell adhesion and proliferation. Their metabolic activity, directly related to their viability, was assessed over 14 days, and it is reported in Figure 8a (fluorescence intensity (FI)). TEN-1 cells proliferate in the GM (growth medium) over the entire time interval of observation, although they show a slight decrease in viability. The same behavior is evident for the random scaffolds. On the other hand, the scaffolds with an aligned structure are able to maintain a constant cell growth over time. Moreover, the doping of CuO and CPP lead to an increase in the cell proliferation greater than that of the scaffolds of PBCA alone. The same trend was observed by means of CLSM (Figure 8b). In fact, the cells preferably adhere onto the aligned scaffolds much more than onto the random ones, and in particular, on the ones doped with both CuO and CPP, where they also assume their typical elongated shape.

### 2.7. Cytocompatibility of Macrophages and Pro-Inflammatory Immune Response

Recent evidence suggests that modulation of inflammation in the early stages following tendon repair may lead to improved healing. It is important to recognize that regulated inflammation is largely beneficial to tissue repair, whereas excessive or persistent inflammation can be damaging and lead to poor clinical outcomes.

For this reason, the anti-inflammatory properties were assessed. The secretion of IL-6 by macrophages stimulated by lipopolysaccharide (LPS) was assessed to evidence whether the extracts from the scaffolds were able to decrease it.

**Figure 9:**
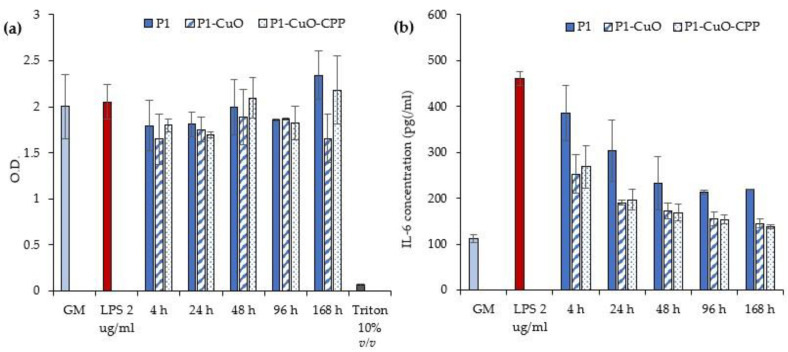
(**a**) Macrophages’ viability after 24 h of contact with LPS and the scaffolds’ extracts at different time intervals. (**b**) IL-6 cytokine concentrations (pg/mL) secreted by the macrophages exposed to different time intervals of scaffolds’ extracts (GM: growth medium; LPS: lipopolysaccharide) (mean values ± SD, *n* = 5). One-way ANOVA, Scheffé test (*p* ≤ 0.05) is reported in the Supplementary Information (SI: Section S1.3.5).

Figure 9a reports the macrophage viability in terms of optical density (OD) after the contact with the scaffold extracts, while Figure 9b reports the IL-6 cytokine concentrations secreted by the macrophages, exposed to the scaffold extracts. The extracts at every time interval are cytocompatible towards macrophages, with a cell viability superimposable to that of the positive control (GM) and the proinflammatory control (LPS), indicating that the scaffolds do not release cytotoxic components. Moreover, the scaffolds doped with CuO lead to a decrease of the IL-6 secretion after 48 h, reaching values significantly lower than those of the LPS. This suggests that the scaffolds decrease the inflammatory response of LPS over time. CuO nanoparticles are conceivably exposed in the cell medium and decorated by GM proteins, forming a protein corona around the nanostructures. This affords them a biological identity and allows them to act as a ligand for the receptors on the M2 anti-inflammatory macrophages, activating them [25].

### 2.8. Antibacterial Activity Evaluation

Metal oxide nanoparticles, such as CuO, have been described as key elements for the control of bacterial growth. In fact, bacteria could develop a resistance against the most used antibiotics/chemotherapeutics. This represents a great threat to human health, and in particular surgical procedures can expose the patients to multidrug-resistant bacteria. However, inorganics, especially in nanostructures, including CuO, are generally effective against local bacterial infections [26].

Table 1 reports the microbicidal effect (ME) (log reduction) evaluated for P1-CuO and P1-CuO-CPP scaffolds at 5 and 24 h of contact with *Escherichia coli* and *Staphylococcus aureus*, used as representative strains of Gram-negative and Gram-positive bacteria, respectively. The scaffolds possess a slight antimicrobial effect against *S. aureus*, while a significant antimicrobial effect is achieved against *E. coli*, since the scaffolds decrease the bioburden by at least 100-fold (a 2-log reduction) after 24 h of contact with the bacteria. In fact, CuO nanoparticles result as mainly effective against Gram-negative bacteria, as highlighted by precedent studies [27,28].

The mechanism of action seems attributable to different pathways, such as the release of CuO^2+^ ions from the nanostructures having a prooxidant effect, and the capability of these nanomaterials to penetrate the bacterial cell membrane. In particular, such nanomaterials are more effective against Gram– bacteria, which possess a membrane mainly formed of peptidoglycans, less firm and easily crossable, while Gram+ bacteria possess a double membrane that is harder to cross [9].

**Figure 10:**
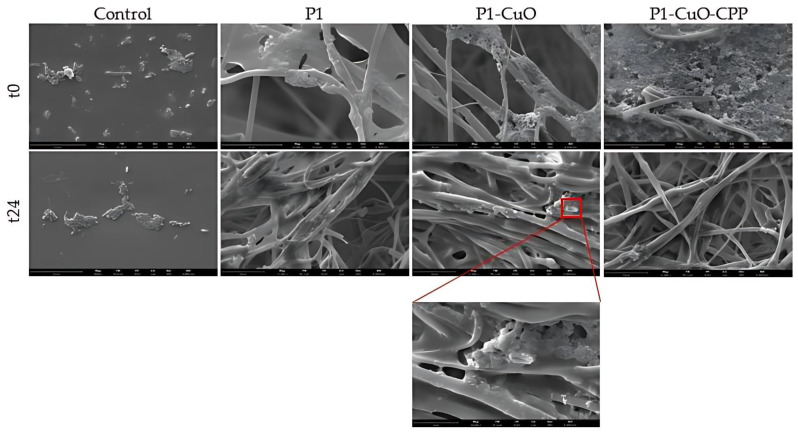
SEM micrographs of *E. coli* colonizing P1, P1-CuO, and P1-CuO-CPP scaffolds after 0 h of contact (t0) and 24 h of contact (t24). A glass slide was used as a control (mean values ± SD, *n* = 4) (scale bar = 15 μm).

To verify the antimicrobial effect and understand if CuO doping impairs the microbial adhesion onto the scaffolds, the SEM analysis of the scaffolds in contact with the *E. coli* at different time intervals (0 h of contact (t0) and 24 h of contact (t24)) was performed (Figure 10). A glass slide was used as a positive control. After 0 h of contact, it is possible to observe *E. coli* in contact with all the scaffolds (undoped and doped) on the fiber surface. Clusters of bacteria were also clearly visible, forming compact colonies with preserved morphology. On the other hand, after 24 h of contact with the scaffolds, the bacteria are no more visible onto the scaffolds, except for P1-CuO fibers, where the bacteria present are not viable, with the cell membrane visibly broken and an altered morphology. These results are in line with the ME evaluation against *E. coli*, confirming that the PBCA scaffolds doped with CuO and CPP could represent an effective tool to avoid bacteria proliferation, and depending on the strain, to also avoid bacteria adhesion and protection of the site of implants from infections after surgery.

## 3. Materials and Methods

### 3.1. Materials

Butyl(2-cyanoacrylate) (BCA) monomer (MW: 153 g/mol) (BLD Pharmatech Ltd., Shanghai, China), CuO nanoparticles (Sigma-Aldrich, Milan, Italy), with particle dimensions < 50 nm, and CPP CE90CPP enzymatically hydrolyzed casein (DMV International, Delhi, NY, USA) with a purity of 26% (*w*/*w*) were used.

### 3.2. Synthesis and Characterization of PBCA

The PBCA was synthesized by emulsion/polymerization of the BCA monomer, as described by Melguizo et al. [29]. This process slightly differs from mini-emulsion–polymerization since the dispersion is not ultrasonicated after stirring. Moreover, it allows some intrinsic advantages, such as a higher rate of polymerization [30,31]. A 0.3 mL acetonic solution (acetone for analysis, ISO-ACS, Carlo Erba Reagents, Milano, Italy) containing 1% *w*/*v* of monomer was prepared in a 1 mL vial. The solution was added dropwise to 3 mL of an aqueous solution containing 0.1 mN HCl (Carlo Erba Reagents, Milano, Italy) and 0.5% *w*/*v* of dextran 500 Da (Pharmacosmos A/S, Roervangsvej, Denmark) under magnetic stirring (3000 rpm). As described by Limouzin et al., dextran acts as a surfactant, both stabilizing the particles and participating in the polymerization. In fact, its counter-ion serves as an initiator of the reaction, and its headgroup forms an ion pair with the propagating species [30].

Polymerization continued for 4 h at room temperature. Then, 3 μL of a 0.1 M NaOH solution (Alfa Aesar by Thermo Fisher Scientific, Kandel, Germany) was added to neutralize the medium, ensuring the total consumption of the monomer. Finally, the solution was heated for 1 h at 60 °C to remove the acetone through evaporation, and the polymer was cleaned by 10 centrifuge cycles (10 min at 10,000 rpm; Sigma 1-14K Refrigerated microcentrifuge, Osterode am Harz, Germany) until the conductivity of the supernatants was ≤10 μS/cm. The supernatant was removed, and the precipitate was frozen at −20 °C and lyophilized for 24 h, at 0.020 mbar (Heto Drywinner sublimator, Analitica De Mori, Italia), to obtain a PBCA powder.

The process *yield* % was then determined as follows:

(1) $$Yield\;\left(\%\right)=\frac{Wf}{Wi}\times 100$$

where *Wi* represents the initial weight of the materials used in the synthesis and *Wf* is the final weight of the PBCA powder.

Proton nuclear magnetic resonance (^1^H NMR) and carbon nuclear magnetic resonance (^13^C NMR) spectra were recorded in deuterated chloroform (CDCl_3_) on a FT-NMR Avance 400 MHz (Bruker, Milan, Italy) spectrometer (equipped with Z-gradient, automatic tuning and shimming, probe BBO 5 mm and BBI 5 mm, auto sampler, Topspin 3.6.1). Chemical shift (δ) was given in ppm relative to the internal standard (TMS).

The molecular weight of the obtained polymer was determined by means of a Litesizer 500 (Anton Paar, Graz, Austria) and the degree of polymerization was calculated as the ration between the polymer molecular weight and the monomer.

### 3.3. Preparation of the Polymeric Blends

Table 2 reports the composition of the polymeric blends used to obtain the corresponding scaffolds. PBCA was solubilized in acetone under magnetic stirring at room temperature. At first, different percentages of PBCA were used to evaluate the influence of the polymer concentration on the fiber morphology. The concentration that allowed the fine formation of nanofibers was selected for the CuO and CPP doping.

### 3.4. Preparation of the Electrospun Scaffolds

Scaffolds were obtained by means of an electrospinning apparatus (STKIT-40, Linari Engineering, Pisa, Italy) equipped with a high-voltage power supply (Razel R99-E 40 kV), a 10 mL syringe with an inox 21G needle, and a volumetric pump (Razel R99-E). A static and flat collector was used to obtain the random scaffolds (namely R P1, R P1-CuO, and R P1-CuO-CPP). The lengthwise-aligned scaffolds (namely A P1, A P1-CuO, and A P1-CuO-CPP) were collected using a cylindrical stainless-steel rotating drum (dimensions: diameter: 3 cm, length: 150 mm). The distance between the needle and the collector was fixed at 15 cm, the voltage at 15 kV, the flow rate at 0.595 cc/h, and the rotation speed at 160 Hz. The relative humidity and the environmental temperature were set at 20% and 25 °C, respectively. All the obtained scaffolds were insoluble in water.

### 3.5. Scaffold Physico-Chemical Characterization

The scaffold morphology was assessed by means of scanning electron microscope (SEM) analysis (Tescan, Mira3XMU, Brno, Czech Republic). The samples were sputtered with graphite. Electrospun nanofiber diameters were assessed by image analysis software (ImageJ, ICY, Institut Pasteur, Paris, France). Moreover, the inclusion of the CuO nanoparticles into the fibrous structure was evaluated by means of SEM-EDX, recording an EDX spectrum, and also by means of transmission electron microscope (TEM) analysis (JEOL JEM-1200 EX II microscope; CCD camera Olympus Mega View G2, with 1376 × 1032-pixel format, Tokyo, Japan; operating HV at 100 kV, magnification 100 k). For this purpose, a thin layer of fiber was electrospun directly onto the TEM grids (formavar/carbon 300-mesh Cu, Agar Scientific, Monterotondo (RM), Italy).

The wettability of the electrospun fibers was assessed with a contact angle meter (DMe-211 Plus; FAMAS software, Kyowa, Osaka, Japan). The droplet shape (0.4 µL of PBS) was captured through the CCD camera at 1 s after the droplet touched the scaffold surface.

### 3.6. Structural Characterization

Fourier-transform infrared spectroscopy (FT-IR) analysis was performed using a JASCO 6200 apparatus (Tokyo, Japan) equipped with a Ge ATR. All analyses were performed from 400 to 4000 cm^−1^ with a resolution of 2 cm^−1^, and the results were processed with Spectra Manager v2 software. The FT-IR spectra were smoothed, and the noise was removed using the Savitzky–Golay filter (OriginPro 2021b, OriginLab Corporation).

The analysis of the nanoscale structure was performed by means of small-angle X-ray scattering (SAXS) at the European Synchrotron Radiation Facility (ESRF, Grenoble, France). Experiments were carried out at the ID02 beamline (https://doi.org/10.15151/ESRF-ES-585935736, accessed on 15 July 2022). Small pieces of scaffolds (0.5 × 0.5 cm) were cut, inserted in 2 mm polycarbonate capillaries (ENKI, Milan, Italy), and fully hydrated with water. Two different sample-to-detector distances (1 and 10 m) have been used to collect the scattered radiation, reaching a wide range of q = 4πsen(ϑ/2)/λ, with ϑ being the scattering angle and λ = 0.1 nm the X-ray wavelength. The intensity spectra as a function of q (0.006 < q < 7.5 nm^−1^) provided information on the mesoscale arrangement of the scaffolds (hundreds of nanometers) and on the local arrangement of polymer chains (nanometer length-scale). Measurements were repeated on different samples, to check for reproducibility.

### 3.7. Mechanical Properties

The mechanical properties of nanofibrous scaffolds were measured using a dynamometer (TA-XT plus, Stable Microsystems, Italy) equipped with a 5.0 kg load cell. Before testing, nanofibrous scaffolds were cut to 30 × 10 mm and the strips were clamped between two tensile grips (A/TG probe), setting an initial distance between the grips of 60.0 mm. Then, the upper grip was moved forward at a constant speed of 5.0 mm/s up to break. The mechanical properties of both random and aligned scaffolds were evaluated in dry and hydrated states, and force at break vs. distance was recorded. Moreover, the elongation and Young’s modulus were calculated [32].

### 3.8. DPPH Radical Scavenging Activity

The free radical scavenging ability (RSA%) of the scaffolds was tested by the DPPH radical scavenging assay. The ability to donate hydrogen atoms was determined by the decolorization of 2,2-diphenyl-1-picrylhydrazyl (DPPH, Sigma-Aldrich, Milan, Italy). In fact, DPPH produces a violet/purple color in methanol solution and turns yellow in the presence of antioxidants. For the time-dependent assay, 10 mg of scaffolds was placed in 2 mL of PBS at 37 °C to simulate the scaffold implant in the lesion bed [33]. At prefixed times, the supernatants were collected to quantify the DPPH activity (direct antioxidant properties). Each sample (1 mL) was mixed with 1 mL of DPPH methanol solution (8 μg/mL) and kept for 30 min in the dark. The absorbance was measured at 515 nm (FLUOstar^®^ Omega, BMG LABTECH, Aylesbury, UK) and the results were expressed as DPPH radical scavenging activity, which was calculated as follows:

(2) $$RSA\;\left(\%\right)=\frac{\left(A0-AS\right)}{A0}\times 100$$

where *A*0 represents the absorbance of the control (DPPH in contact with water), and *AS* is the absorbance of the scaffolds after the contact with DPPH [34].

### 3.9. Cell Adhesion and Proliferation

Proliferation and cell viability were carried out using normal human tenocytes (TEN-1) (1st–5th passages, ZenBio, Durham, NC, USA). TEN-1 were cultured in collagen (rat tail collagen coating solution, Cell Applications, Italy) coated flasks, using tenocyte growth medium (ZenBio, Durham, NC, USA) supplemented with 10% *v*/*v* fetal bovine serum (FBS, Euroclone, Milan, Italy) and with 200 IU/mL of penicillin/0.2 mg/mL of streptomycin (Sigma-Aldrich, Milan, Italy). They were grown in an incubator (CO_2_ Incubator, PBI International, Milano, Italy) at 37 °C in a 5% CO_2_ atmosphere with 95% relative humidity (RH).

Each scaffold (5 mm diameter, 0.2 mm thickness) was sterilized by UV radiation for 15 min and placed in a 96-well plate to perfectly cover the bottom. TEN-1 were seeded onto the scaffolds at a density of 20 × 10^3^ cells/well and re-incubated. TEN-1 grown in standard conditions (growth medium, GM) were considered as a positive control. After 7 and 14 days of contact with the scaffolds, the medium was removed and 10% (*v*/*v*) Alamar Blue (AlamarBlue HS cell viability reagent, Invitrogen, Thermo Fisher, Monza, Italy) was diluted with the appropriate medium and added in the wells (100 µL). After 3 h of incubation in the dark at 37 °C, the Alamar Blue solution was collected from the wells and transferred to a new plate. Each well was then refilled with the culture medium and left in culture again. Alamar Blue fluorescence was recorded using a microplate reader (FLUOstar^®^ Omega, BMG LABTECH, Aylesbury, UK), with λex = 530 nm and λem = 590 nm. Cell viability was expressed as fluorescence intensity (FI). 

The cell morphology after 14 days of contact with the scaffolds was investigated using CLSM after nuclei and cytoskeleton staining. Cells grown onto the scaffolds were fixed using a 3% (*v*/*v*) glutaraldehyde solution in PBS for 2 h at room temperature. The substrates were then washed three times with PBS. The cellular cytoskeleton was stained with FITC Atto 488 phalloidin (green, Sigma-Aldrich, Milan, Italy; 50 μL at 20 μg/mL in PBS in each well, contact time 40 min), and the cell nuclei were then stained with propidium iodide (red, Sigma-Aldrich, Milano, Italy; 50 µL/sample at 25 µg/mL in PBS in each well, contact time 2 min). Scaffolds were placed onto microscope slides and imaged using a Confocal Laser Scanning Microscope (CLSM, Leica TCS SP2, Leica Microsystems, Buccinasco (MI), Italy), with λex = 535 nm and λem = 617 nm for propidium iodide, and λex = 501 nm and λem = 523 nm for FITC-phalloidin. The acquired images were processed with software (Leica Microsystem, Buccinasco (MI), Italy).

### 3.10. Cytocompatibility of Macrophages and Pro-Inflammatory Immune Response

The hMoCD14 + -PB-c cell line, human CD14+ monocytes derived from peripheral blood (Carlo Erba, Italy), was cultured in mononuclear cell medium (Carlo Erba, Italy) supplemented with 10% fetal bovine serum (FBS, Euroclone, Italy), and with 200 IU/mL of penicillin/0.2 mg/mL of streptomycin (Sigma-Aldrich, Italy), kept at 37 °C in a 5% CO_2_ atmosphere with 95% relative humidity (RH). Each scaffold was sterilized by UV radiation for 15 min and then incubated in a serum-free DMEM medium (Dulbecco’s Modified Eagle’s Medium, PromoCell, WVR, Italy) for 24 h to produce extraction media of 10 mg/mL. hMoCD14 + -PB-c were seeded 20 × 10^3^ cells/well in a 24-well plate and incubated for 24 h with 100 nM for 2 × 10^4^ cells of phorbol 12-myristate-13-acetate (PMA, Sigma-Aldrich, Italy) to allow differentiation. After differentiation, extracted media were placed in the wells and incubated for 24 h. The cytocompatibility of the scaffold extracts was assessed using the MTT (3-(4,5-dimethylthiazol-2-yl)-2,5-diphenyltetrazolium bromide) assay. The medium in each well was removed and 100 μL of MTT solution at 1 mg/mL in DMEM w/o phenol red (Sigma-Aldrich, Milan, Italy) was added. Then, the cell substrates were placed at 37 °C for 3 h in an incubator, and finally, MTT solution was removed and 100 μL of isopropanol (Carlo Erba, Italy) was added, and the absorbance was read using the FLUOstar^®^ Omega Microplate Reader (BMG LABTECH, Italy) at λ = 570 nm (with reference λ = 690 nm). IL-6 pro-inflammatory cytokine was assayed to evaluate the proinflammatory immune response using the ELISA kit (Thermo Fisher, Italy). Supernatants were collected from the cultures after 24 h of treatment with the scaffolds and the cytokine secretion by macrophages was assayed at 450 nm, with 570 nm as the reference wavelength. The method was linear in the concentration range from 7.8 to 500 pg/mL, with the R^2^ always higher than 0.995. Lipopolysaccharide (LPS, 10 μg/mL for 24 h) was used as a positive control [35].

### 3.11. Antibacterial Activity Evaluation

The scaffold antibacterial activity was tested against two different microorganisms by measuring the microbicidal effect (ME): *Staphylococcus aureus* ATCC 6538 and *Escherichia coli* ATCC 10356 were used as representative strains of Gram-positive and Gram-negative bacteria, respectively. These bacteria were grown overnight at 37 °C in Tryptone Soya Broth (Oxoid, Basingstoke, Hampshire, UK). Bacterial cultures were centrifuged at 3000 rpm for 20 min to separate cells from the broth and then suspended in sterile water. The optical density of the microbial suspensions was adjusted to A = 0.3 (wavelength: 650 nm), which corresponds to a number of cells of: 1 × 10^7^–1 × 10^8^ CFU/mL. To measure the ME of the scaffolds, microbial suspensions (10 µL) were placed on glass slides (26 × 76 mm) and covered with the scaffolds (20 × 20 mm). This glass–scaffold system was maintained in a humid environment (with 1 mL of PBS in a Falcon test-tube) at room temperature for 5 and 24 h. The same setup was used with the undoped scaffolds (P1) as a control. After the two contact times, a volume of 9 mL of sterile water was added in each test-tube, gently shaking to obtain the detachment of the scaffold from the slides. Microbial suspensions were then placed in Tryptone Soya Agar (Oxoid; Basingstoke, Hampshire, UK) and bacterial colonies were enumerated after incubation at 37 °C for 24 h [36]. The decimal-log reduction rate, the microbicidal effect (*ME* value), was calculated for each test organism and contact time according to the following equation [37]:

(3) ME=logNc−logNd

where *Nc* is the number of CFU of the control microbial suspension and *Nd* is the number of CFU of the microbial suspension in the presence of the scaffolds with CuO nanoparticles and CPP.

The antibacterial activity was also evaluated by SEM analysis, to explore the initial bacterial adhesion and progressive spreading and colonization onto the electrospun scaffolds exposed to *Escherichia coli*. Here, 10 µL of the microbial suspension was placed onto the scaffolds and covered with microscope glasses to allow the exposure of the whole surface to the bacteria. A glass slide was used as a positive control. At different time intervals of incubation (0 and 24 h), the scaffolds were put in contact with 1 mL of 3% (*v*/*v*) glutaraldehyde solution in PBS for 2 h at room temperature for bacterial fixation. Once dried, the scaffolds were mounted on SEM supports and sputtered with 5 nm of gold.

### 3.12. Statistical Analysis

Statistical analyses were performed using the Astatsa statistical calculator. One-way analysis of variance (ANOVA) was followed by the Scheffé test for post-hoc comparisons. Here, *p* < 0.05 was considered significant.

## 4. Conclusions

Nanofibrous scaffolds based on PBCA and loaded with CuO and CPP were successfully prepared by means of the electrospinning technique. Moreover, it was possible to obtain fibers in an aligned conformation able to mimic the tendon fascicles. The obtained scaffolds are characterized for their morphology, showing nanometric dimensions and an orderly structural organization.

The evaluation of the mechanical properties highlights that CuO and CPP determine an increase in the mechanical performance of the scaffolds; moreover, the aligned conformation leads to a significant increase of these properties with respect to the fibers in the random conformation. Furthermore, the CuO induces a significant increase in the hydrophobic characteristics of the scaffolds by decreasing their wettability, which could cause a slowdown in the degradation rate of the scaffolds in vivo, ensuring adequate support to the injured tissue during the entire healing process. Moreover, the scaffolds loaded with CuO also show antioxidant properties that could protect the site of inflammation from severe tissue damage and impair cell proliferation and migration.

The scaffolds prove to be biocompatible towards tenocytes and able to promote cell adhesion and proliferation. In particular, CuO and CPP seem to have a synergic effect on cell adhesion and proliferation. The scaffolds are also characterized by remarkable anti-inflammatory properties that could represent an interesting tool to overcome the poor clinical outcomes related to excessive inflammation.

The antibacterial activity evaluation demonstrates that the CuO-doped scaffolds possess a significant antimicrobial effect against *E. coli*, since they are able to decrease the bioburden by at least 100-fold (a 2-log reduction) after 24 h of contact, resulting as mainly effective against Gram-negative bacteria. The results were confirmed with SEM analysis, which highlights that the bacteria after 24 h are not present on the scaffold surface, except for P1-CuO fibers, where the bacteria are not viable, presenting the cell membrane as visibly broken.

In conclusion, scaffolds based on PBCA and doped with CuO and CPP deserve particular attention as enhancers of the tendon tissue regeneration. Further investigation of the scaffold efficacy in vivo will assess their capability for enhancing the tendon ECM restoration, and eventually accelerate their translation to the clinic.

## Figures and Tables

**Table 1:** Microbicidal effect (ME) evaluated for P1-CuO and P1-CuO-CPP scaffolds against *E. coli* and *S. aureus* at 5 and 24 h of contact. One-way ANOVA, Scheffé test (*p* ≤ 0.05) is reported in the Supplementary Information (SI: Section S1.3.6).

	*E. coli*		*S. aureus*	
	5 h	24 h	5 h	24 h
P1-CuO	0.0	2.99 ± 0.09	0.04 ± 0.03	1.01 ± 0.27
P1-CuO-CPP	0.0	1.72 ± 0.31	0.0	0.59 ± 0.27

**Table 2:** Qualitative–quantitative composition of the polymeric blends.

Blend	PBCA % (*w*/*w*)	CuO % (*w*/*w*)	CPP % (*w*/*w*)
**P1**	20		
**P2**	23		
**P3**	30		
**P1-CuO**	20	0.01	
**P1-CuO-CPP**	20	0.01	0.25

## Data Availability

Not applicable.

## Supplementary Materials

The following supporting information can be downloaded at: https://www.mdpi.com/article/10.3390/ijms24043172/s1.
