# Supplementary material for: Electrospun Scaffolds Based on Poly(butyl cyanoacrylate) for Tendon Tissue Engineering
Source: Int J Mol Sci. 2023 Feb 6;24(4):3172. doi: 10.3390/ijms24043172 (PMC9960733; doi:10.3390/ijms24043172)
Supplement: Supplementary file 1 [file ijms-24-03172-s001.zip › ijms-2156314-supplementary.pdf]

Supplementary Information

Electrospun Scaffolds Based on Poly(butyl cyanoacrylate) for Tendon Tissue Engineering

Eleonora Bianchi [1], Barbara Vigani [1], Marco Ruggeri [1], Elena Del Favero [2], Caterina Ricci [2], Pietro Grisoli [1], Anita Ferraretto [3,4], Silvia Rossi [1], César Viseras [5], Giuseppina Sandri [1*]

[1] Department of Drug Sciences, University of Pavia, Viale Taramelli 12, 27100, Pavia, Italy
[2] Department of Medical Biotechnology and Translational Medicine, University of Milan, LITA Viale Fratelli Cervi 93, 20090 Segrate, Italy
[3] IRCCS Istituto Ortopedico Galeazzi, Laboratory of Experimental Biochemistry & Molecular Biology, Via R. Galeazzi 4, 20161 Milan, Italy
[4] Dipartimento di Scienze Biomediche per la Salute, Università degli Studi di Milano, LITA, Via Fratelli Cervi 93, 20090 Segrate, Italy
[5] Department of Pharmacy and Pharmaceutical Technology, Faculty of Pharmacy, University of Granada, Campus of Cartuja s/n, Granada, 18071, Spain
* Correspondence: g.sandri@unipv.it

1. Results and Discussion

1.1. Scaffolds Physico-Chemical Characterization

In Figure S1 the SEM microphotographs of the P1, and P2 fibers are shown. The PBCA optimal concentration to obtain fine formation of nanofibers with the homogeneous morphology was 20 % w/w. In particular, it was possible to observe that the PBCA concentration affected the fibers morphology. In fact, the concentration increase led to the formation of irregular fibers, and knots. Moreover, the P3 blend, containing the highest polymer concentration, did not even allow to obtain a regular production of fibers. On the other hand, the lowest polymer concentration led to the formation of uniform fibers with a smooth surface and nanometric dimensions.

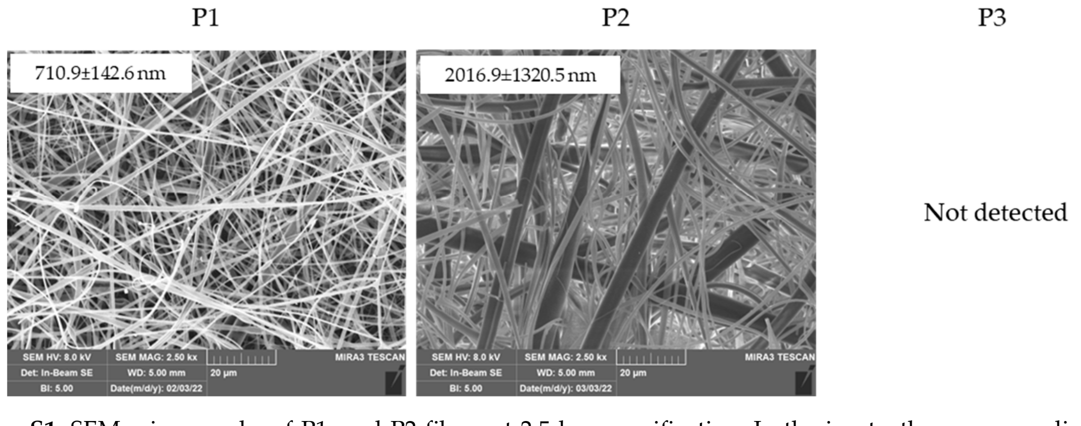

Figure S1. SEM micrographs of P1, and P2 fibers at 2.5 kx magnification. In the insets, the corresponding dimensional analysis is shown (mean values ± s.d.; n=30).

1.2. Structural Characterization

Figure S2 reports the FTIR spectra of the raw materials that compose the scaffolds.

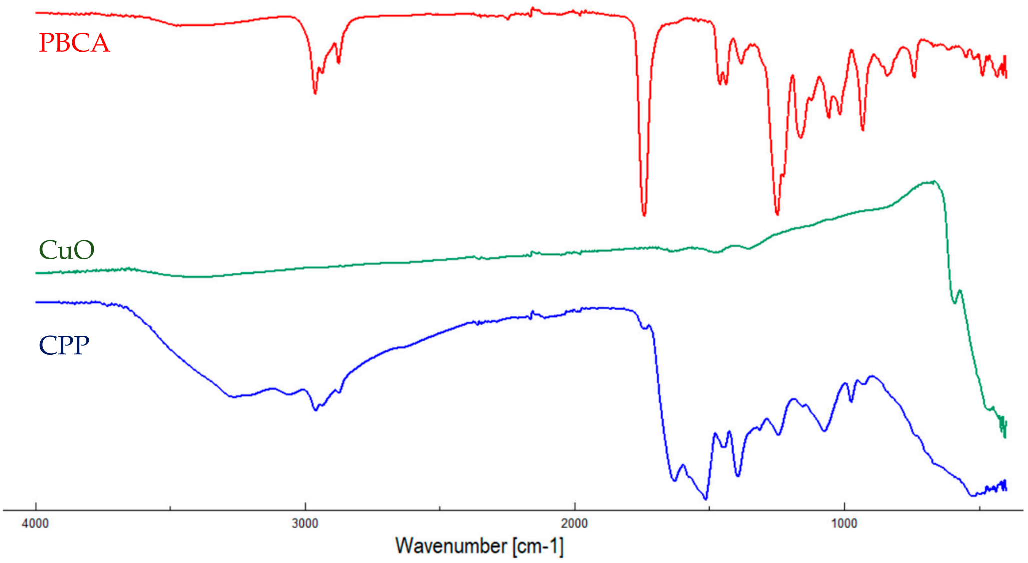

Figure S2. FTIR spectra of the scaffolds raw materials.

Figure S3 reports the SAXS spectra of the raw materials used as doping components.

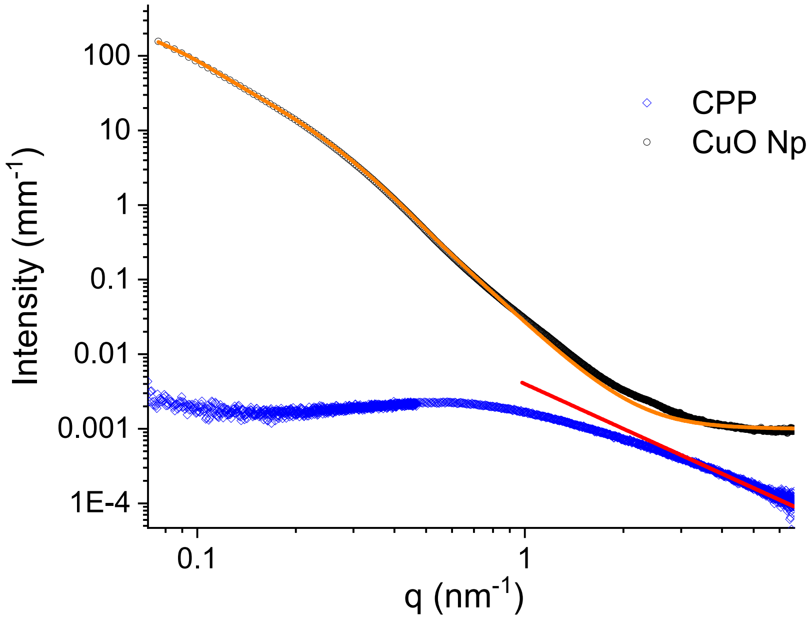

Figure S3. Small angle x-ray scattering (SAXS) spectra of copper oxide nanoparticles CuO Np (black dots) and caseinphosphopeptides CPP (blue diamonds) in aqueous dispersion at T = 25 °C. The orange line is the fitting curve, obtained with a rod-like model for CuO nanoparticles (short size = 10 nm, long size = 70 nm). The red line shows the intensity decay behaviour of CPP in the high-q region, corresponding to distances shorter than 1.7 nm: $I(q) = q^{-2}$ as expected for gaussian polymer chains. The inflection of the intensity in the low-q region indicates that CPPs peptides are in an interacting regime when observed at 2.5 mg/ml.

1.3. Statistical Analysis

1.3.1. Scaffolds Physico-Chemical Characterization

The statistical analysis of Figure 4 (scaffolds wettability) is here reported:

R P1 vs R P1-CuO: $p < 0.01$; R P1 vs R P1-CuO-CPP: $p < 0.01$; R P1 vs A P1: $p < 0.01$; R P1 vs A P1-CuO: $p < 0.01$; R P1 vs A P1-CuO-CPP: $p < 0.01$; R P1-CuO vs R P1-CuO-CPP: p = 0.8999; R P1-CuO vs A P1: p = 0.7938; R P1-CuO vs A P1-CuO: $p < 0.01$; R P1-CuO vs A P1-CuO-CPP: p = 0.1777; R P1-CuO-CPP vs A P1: p = 0.8999; R P1-CuO-CPP vs A P1-CuO: $p < 0.01$; R P1-CuO-CPP vs A P1-CuO-CPP: p = 0.3901; A P1 vs A P1-CuO: $p < 0.01$; A P1 vs A P1-CuO-CPP: p = 0.7360; A P1-CuO vs A P1-CuO-CPP: p = 0.0503.

1.3.2. Mechanical Properties Evaluation

The statistical analysis of Figure 6 (scaffolds mechanical properties) is here reported:

(a) (Fmax) R P1 vs R P1-CuO: p = 0.7999; R P1 vs R P1-CuO-CPP: $p < 0.01$; R P1 vs A P1: $p < 0.01$; R P1-CuO vs R P1-CuO-CPP: $p < 0.01$; R P1-CuO vs A P1-CuO: $p < 0.01$; R P1-CuO-CPP vs A P1-CuO-CPP: p = 0.7070; A P1 vs A P1-CuO: p = 0.0768; A P1 vs A P1-CuO-CPP: $p < 0.01$; A P1 CuO vs A P1-CuO-CPP: p = 0.6960; (Elongation %) R P1 vs R P1-CuO: p = 0.2650; R P1 vs R P1-CuO-CPP: p = 0.2392; R P1 vs A P1: p = 0.9273; R P1-CuO vs R P1-CuO-CPP: p = 0.9998; R P1-CuO vs A P1-CuO: p = 0.8856; R P1-CuO-CPP vs A P1-CuO-CPP: $p < 0.01$; A P1 vs A P1-CuO: p = 0.0171; A P1 vs A P1-CuO-CPP: p = 0.5451; A P1 CuO vs A P1-CuO-CPP: p = 0.0923; (Young's modulus) R P1 vs R P1-CuO: p = 0.1149; R P1 vs R P1-CuO-CPP: $p < 0.01$; R P1 vs A P1: p 0.01; R P1-CuO vs R P1-CuO-CPP: $p < 0.01$; R P1-CuO vs A P1-CuO: p = 0.0331; R P1-CuO-CPP vs A P1-CuO-CPP: $p < 0.01$; A P1 vs A P1-CuO: p = 0.4320; A P1 vs A P1-CuO-CPP: p = 0.5602; A P1 CuO vs A P1-CuO-CPP: p = 0.9719; (b) (Fmax) R P1 vs R P1-CuO: p = 0.9925; R P1 vs R P1-CuO-CPP: $p < 0.01$; R P1 vs A P1: $p < 0.01$; R P1-CuO vs R P1-CuO-CPP: $p < 0.01$; R P1-CuO vs A P1-CuO: $p < 0.01$; R P1-CuO-CPP vs A P1-CuO-CPP: p = 0.5994; A P1 vs A P1-CuO: p = 0.0424; A P1 vs A P1-CuO-CPP: $p < 0.01$; A P1 CuO vs A P1-CuO-CPP: p = 0.5714; (Elongation %) R P1 vs R P1-CuO: p = 0.1694; R P1 vs R P1-CuO-CPP: p = 0.4526; R P1 vs A P1: p = 0.4588; R P1-CuO vs R P1-CuO-CPP: p = 0.1394; R P1-CuO vs A P1-CuO: p = 0.5151; R P1-CuO-CPP vs A P1-CuO-CPP: $p < 0.01$; A P1 vs A P1-CuO: p = 0.0254; A P1 vs A P1-CuO-CPP: p = 0.8187; A P1 CuO vs A P1-CuO-CPP: p = 0.0118; (Young's modulus) R P1 vs R P1-CuO: p = 0.8464; R P1 vs R P1-CuO-CPP: $p < 0.01$; R P1 vs A P1: p = 0.0626; R P1-CuO vs R P1-CuO-CPP: $p < 0.01$; R P1-CuO vs A P1-CuO: p = 0.0350; R P1-CuO-CPP vs A P1-CuO-CPP: $p < 0.01$; A P1 vs A P1-CuO: p = 0.9978; A P1 vs A P1-CuO-CPP: p = 0.5602; A P1 CuO vs A P1-CuO-CPP: p = 0.5972.

1.3.3. DPPH Radical Scavenging Activity

The statistical analysis of Figure 7 (scaffolds radical scavenging activity) is here reported:

(1h) P1 vs P1-CuO: p = 0.4059; P1 vs P1-CuO-CPP: p = 0.6345; P1 vs CuO: p = 0.8079; P1-CuO vs P1-CuO-CPP: p = 0.9719; P1-CuO vs CuO: p = 0.8750; P1-CuO-CPP vs CuO: p = 0.9880; (4h) P1 vs P1-CuO: p = 0.2742; P1 vs P1-CuO-CPP: p = 0.0288; P1 vs CuO: p = 0.4483; P1-CuO vs P1-CuO-CPP: p = 0.4292; P1-CuO vs CuO: p = 0.9767; P1-CuO-CPP vs CuO: p = 0.2608; (8h) P1 vs P1-CuO: p = 0.0105; P1 vs P1-CuO-CPP: $p < 0.01$; P1 vs CuO: p = 0.0465; P1-CuO vs P1-CuO-CPP: p = 0.0874; P1-CuO vs CuO: p = 0.7140; P1-CuO-CPP vs CuO: p = 0.3258; (24h) P1 vs P1-CuO: p = 0.1746; P1 vs P1-CuO-CPP: p = 0.1663; P1 vs CuO: p = 0.2983; P1-CuO vs P1-CuO-CPP: p = 0.9999; P1-CuO vs CuO: p = 0.9772; P1-CuO-CPP vs CuO: p = 0.9710; (48h) P1 vs P1-CuO: p = 0.0223; P1 vs P1-CuO-CPP: p = 0.0868; P1 vs CuO: p = 0.0560; P1-CuO vs P1-CuO-CPP: p = 0.7782; P1-CuO vs CuO: p = 0.9132; P1-CuO-CPP vs CuO: p = 0.9895.

1.3.4. Cell Adhesion and Proliferation

The statistical analysis of Figure 8 (Alamar blue assay) is here reported:

(7 days) GM vs R P1: $p < 0.01$; GM vs R P1-CuO: $p < 0.01$; GM vs R P1-CuO-CPP: $p < 0.01$; GM vs A P1: $p < 0.01$; GM vs A P1-CuO: p = 0.0271; GM vs A P1-CuO-CPP: p = 0.2336; R P1 vs R P1-CuO: p = 0.9997; R P1 vs R P1-CuO-CPP: p = 0.9999; R P1 vs A P1: p = 0.9995; R P1 vs A P1-CuO: p = 0.8300; R P1 vs A P1-CuO-CPP: p = 0.2779; R P1-CuO vs R P1-CuO-CPP: p = 0.9990; R P1-CuO vs A P1: p = 0.9840; R P1-CuO vs A P1-CuO: p = 0.9611; R-CuO P1 vs A P1-CuO-CPP: p = 0.5024; R P1-CuO-CPP vs A P1: p = 0.9999; R P1-CuO-CPP vs A P1-CuO: p = 0.7771; R-CuO-CPP P1 vs A P1-CuO-CPP: p = 0.2308; A P1 vs A P1-CuO: p = 0.5750; A P1 vs A P1-CuO-CPP: p = 0.1180; A P1-CuO vs A P1-CuO-CPP: p = 0.9694; (14 days) GM vs R P1: $p < 0.01$; GM vs R P1-CuO: $p < 0.01$; GM vs R P1-CuO-CPP: p = 0.0356; GM vs A P1: p = 0.1924; GM vs A P1-CuO: p = 0.9783; GM vs A P1-CuO-CPP: p = 0.7814; R P1 vs R P1-CuO: p = 0.8280; R P1 vs R P1-CuO-CPP: p = 0.0520; R P1 vs A P1: $p < 0.01$; R P1 vs A P1-CuO: $p < 0.01$; R P1 vs A P1-CuO-CPP: $p < 0.01$; R P1-CuO vs R P1-CuO-CPP: p = 0.6354; R P1-CuO vs A P1: p = 0.2176; R P1-CuO vs A P1-CuO: $p < 0.01$; R-CuO P1 vs A P1-CuO-CPP: $p < 0.01$; R P1-CuO-CPP vs A P1: p = 0.9914; R P1-CuO-CPP vs A P1-CuO: p = 0.2518; R-CuO-CPP P1 vs A P1-CuO-CPP: $p < 0.01$; A P1 vs A P1-CuO: p = 0.6849; A P1 vs A P1-CuO-CPP: $p < 0.01$; A P1-CuO vs A P1-CuO-CPP: p = 0.2623.

1.3.5. Cytocompatibility of Macrophages and Pro-Inflammatory Immune Response

The statistical analysis of Figure 9 (Macrophages viability and IL-6 cytokine concentration) is here reported:

(a) GM vs LPS: $p = 0.9999$; GM vs P1 4h: $p = 0.9969$; GM vs P1-CuO 4h: $p = 0.9285$; GM vs P1-CuO-CPP 4h: $p = 0.9982$; GM vs P1 24h: $p = 0.9669$; GM vs P1-CuO 24h: $p = 0.9657$; GM vs P1-CuO-CPP 24h: $p = 0.9828$; GM vs P1 48h: $p = 0.8416$; GM vs P1-CuO 48h: $p = 0.9851$; GM vs P1-CuO-CPP 48h: $p = 0.9878$; GM vs P1 96h: $p = 0.9086$; GM vs P1-CuO 96h: $p = 0.9062$; GM vs P1-CuO-CPP 96h: $p = 0.9995$; GM vs P1 168h: $p = 0.9990$; GM vs P1-CuO 168h: $p = 0.9999$; GM vs P1-CuO-CPP 168h: $p = 0.9994$; Triton vs LPS: p 0.01; Triton vs P1 4h: $p < 0.01$; Triton vs P1-CuO 4h: $p < 0.01$; Triton vs P1-CuO-CPP 4h: $p < 0.01$; Triton vs P1 24h: $p < 0.01$; Triton vs P1-CuO 24h: $p < 0.01$; Triton vs P1-CuO-CPP 24h: $p < 0.01$; Triton vs P1 48h: $p < 0.01$; Triton vs P1-CuO 48h: $p < 0.01$; Triton vs P1-CuO-CPP 48h: $p < 0.01$; Triton vs P1 96h: $p < 0.01$; Triton vs P1-CuO 96h: $p < 0.01$; Triton vs P1-CuO-CPP 96h: $p < 0.01$; Triton vs P1 168h: $p < 0.01$; Triton vs P1-CuO 168h: $p < 0.01$; Triton vs P1-CuO-CPP 168h: $p < 0.01$; LPS vs P1 4h: $p = 0.9819$; LPS vs P1-CuO 4h: $p = 0.9872$; LPS vs P1-CuO-CPP 4h: $p = 0.8358$; LPS vs P1 24h: $p = 0.9844$; LPS vs P1-CuO 24h: $p = 0.9048$; LPS vs P1-CuO-CPP 24h: $p = 0.9024$; LPS vs P1 48h: $p = 0.9999$; LPS vs P1-CuO 48h: $p = 1.0000$; LPS vs P1-CuO-CPP 48h: $p = 0.9999$; LPS vs P1 96h: $p = 0.9581$; LPS vs P1-CuO 96h: $p = 0.9990$; LPS vs P1-CuO-CPP 96h: $p = 0.9682$; LPS vs P1 168h: $p = 0.8429$; LPS vs P1-CuO 168h: $p = 0.8396$; LPS vs P1-CuO-CPP 168h: $p = 1.0000$; (b) GM vs LPS: $p < 0.01$; GM vs P1 4h: $p < 0.01$; GM vs P1-CuO 4h: $p < 0.01$; GM vs P1-CuO-CPP 4h: $p < 0.01$; GM vs P1 24h: $p < 0.01$; GM vs P1-CuO 24h: $p =0.0856$; GM vs P1-CuO-CPP 24h: $p = 0.1618$; GM vs P1 48h: $p = 0.0265$; GM vs P1-CuO 48h: $p = 0.2999$; GM vs P1-CuO-CPP 48h: $p = 0.6024$; GM vs P1 96h: $p = 0.0374$; GM vs P1-CuO 96h: $p = 0.6855$; GM vs P1-CuO-CPP 96h: $p = 0.8739$; GM vs P1 168h: $p = 0.0421$; GM vs P1-CuO 168h: $p = 0.8874$; GM vs P1-CuO-CPP 168h: $p = 0.9901$; LPS vs P1 4h: $p = 0.4433$; LPS vs P1-CuO 4h: $p < 0.01$; LPS vs P1-CuO-CPP 4h: $p < 0.01$; LPS vs P1 24h: $p < 0.01$; LPS vs P1-CuO 24h: $p < 0.01$; LPS vs P1-CuO-CPP 24h: $p < 0.01$; LPS vs P1 48h: $p < 0.01$; LPS vs P1-CuO 48h: $p < 0.01$; LPS vs P1-CuO-CPP 48h: $p < 0.01$; LPS vs P1 96h: $p < 0.01$; LPS vs P1-CuO 96h: $p < 0.01$; LPS vs P1-CuO-CPP 96h: $p < 0.01$; LPS vs P1 168h: $p < 0.01$; LPS vs P1-CuO 168h: $p < 0.01$; LPS vs P1-CuO-CPP 168h: $p < 0.01$; P1 4h vs P1 24h: $p = 0.3432$; P1 4h vs P1 48h: $p < 0.01$; P1 4h vs P1 96h: $p < 0.01$; P1 4h vs P1 168h: $p < 0.01$; P1 24h vs P1 48h: $p = 0.5373$; P1 24h vs P1 96h: $p = 0.2468$; P1 24h vs P1 168h: $p = 0.3204$; P1 48h vs P1 96h: $p = 0.9991$; P1 48h vs P1 168h: $p = 0.9999$; P1 96 h vs P1 168h: $p = 1.0000$; P1-CuO 4h vs P1-CuO 24h: $p = 0.2649$; P1-CuO 4h vs P1-CuO 48h: $p = 0.0734$; P1-CuO 4h vs P1-CuO 96h: $p = 0.0186$; P1-CuO 4h vs P1-CuO 168h: $p < 0.01$; P1-CuO 24h vs P1-CuO 48h: $p = 0.9958$; P1-CuO 24h vs P1-CuO 96h: $p = 0.8547$; P1-CuO 24h vs P1-CuO 168h: $p = 0.6373$; P1-CuO 48h vs P1-CuO 96h: $p = 0.9970$; P1-CuO 48h vs P1-CuO 168h: $p = 0.9574$; P1-CuO 96 h vs P1-CuO 168h: $p = 0.9999$; P1-CuO-CPP 4h vs P1-CuO-CPP 24h: $p = 0.3293$; P1-CuO-CPP 4h vs P1-CuO-CPP 48h: $p = 0.0659$; P1-CuO-CPP 4h vs P1-CuO-CPP 96h: $p = 0.0239$; P1-CuO-CPP 4h vs P1-CuO-CPP 168h: p 0.01 ; P1-CuO-CPP 24h vs P1-CuO-CPP 48h: $p = 0.9824$; P1-CuO-CPP 24h vs P1-CuO-CPP 96h: $p = 0.8458$; P1-CuO-CPP 24h vs P1-CuO-CPP 168h: $p = 0.5489$; P1-CuO-CPP 48h vs P1-CuO-CPP 96h: $p = 0.9996$; P1-CuO-CPP 48h vs P1-CuO-CPP 168h: $p = 0.9673$; P1-CuO-CPP 96 h vs P1-CuO-CPP 168h: $p = 0.9994$; P1 4h vs P1-CuO 4h: $p = 0.0248$; P1 4h vs P1-CuO-CPP 4h: $p = 0.0200$; P1-CuO 4h vs P1-CuO-CPP 4h: $p = 0.8999$; P1 24h vs P1-CuO 24h: $p = 0.0112$; P1 24h vs P1-CuO-CPP 24h: $p = 0.0148$; P1-CuO 24h vs P1-CuO-CPP 24h: $p = 0.9646$; P1 48h vs P1-CuO 48h: $p = 0.0937$; P1 48h vs P1-CuO-CPP 48h: $p = 0.0784$; P1-CuO 48h vs P1-CuO-CPP 48h: $p = 0.9893$; P1 96h vs P1-CuO 96h: $p = 0.0254$; P1 96h vs P1-CuO-CPP 96h: $p = 0.0232$; P1-CuO 96h vs P1-CuO-CPP 96h: $p = 0.9967$; P1 168h vs P1-CuO 168h: $p < 0.01$; P1 168h vs P1-CuO-CPP 168h: $p < 0.01$; P1-CuO 168h vs P1-CuO-CPP 168h: $p = 0.2207$.

1.3.6. Antibacterial Activity Evaluation

The statistical analysis of Table 1 (Microbicidal effect (ME)) is here reported:

E. coli: P1-CuO 5h vs P1-CuO 24 h: $p < 0.01$; P1-CuO-CPP 5h vs P1-CuO-CPP 24 h: $p < 0.01$; *S. aureus*: P1-CuO 5h vs P1-CuO 24 h: $p < 0.01$; P1-CuO-CPP 5h vs P1-CuO-CPP 24 h: $p = 0.0487$.
